# Active carbon-based waste packaging materials for uranium sorption from aqueous solution

**DOI:** 10.1007/s11356-023-27269-7

**Published:** 2023-05-11

**Authors:** Amir Elzoghby, Hager Fahmy, Mohamed Taha, Saber Ibrahim

**Affiliations:** 1 Nuclear Materials Authority, El Maddi, P.O. Box 530, Cairo, Egypt; 2 Department of Advertising, Printing and, Publishing, Faculty of Applied Arts, Benha University, Qalubia, 13518 Egypt; 3 Packaging Materials Department, National Research Centre, Elbehouth Street 33, Cairo, 12622 Dokki Egypt

**Keywords:** Active carbon, Add-value waste, Packaging materials, Uranium sorption

## Abstract

Waste (packaging plastic and industrial water) accumulation is one of the great global challenges over the world. Combining waste recycling science and water treatment knowledge are fascinating as applied sciences add value to the safe disposal of waste plastic packaging materials and wastewater. Active carbons (ACs) are prepared from polyethylene terephthalate (PET) at two pyrolysis temperatures (i.e. 450 and 500 °C) and compressed in well-defined designed molds to form cylinder shapes as applied in industry. Particle size (817 and 1074 nm), zeta potential (− 7.17 and − 25.6 mV), surface area (544 and 632 m2/g), and topography of prepared ACs were investigated and discussed. Zeta potential exhibited nice dispersion in accordance to charge value and surficial SEM images prove space hole filling with adsorbed materials after treatment. The prepared activated carbon sorbents have been applied for the removal of radioactive elements from wastewater. The displayed data declare that both sorbents have the same sorption performance, whereas the uranium sorption process using both sorbents is obeyed to pseudo-second-order kinetic model and Langmuir isotherm model. Nevertheless, it is worth noting that the prepared AC at a pyrolysis temperature of 500 °C exhibits higher sorption capacity (38.9 mg g^−1^) than that prepared at lower temperature, i.e., 450 °C (36.2 mg g^−1^) which indicates that the increase in pyrolysis temperature improves the sorption characteristics of the yield-activated carbon.

**Supplementary Information:**

The online version contains supplementary material available at 10.1007/s11356-023-27269-7.

## Introduction

Consumable polymeric materials have extremely increased during last decades. Yearly consumption for each person over the world is about 117 kg; about 367 million metric tons in 2020 of plastics are consumed (Sid et al. 2021). Waste packaging materials are shared by 90 million metric tons of plastic waste (Sid et al. 2021). Over the world, more than one million drinking bottles based on plastic packaging materials are bought every minute. Additionally, 5 trillion one-use plastic packaging bags are used universal per year (Meawad and Ibrahim 2019). In overall, 50% of consumed packaging plastic material is planned to be used one time and then thrown as waste plastic (Meawad and Ibrahim 2019). The recycling of plastic waste-based packaging materials is targeted for global governments, United Nations associations and funding research institutes.

PET is commonly cast-off in packaging for various applications (Meawad and Ibrahim 2019; Sid et al. 2021). PET as a polymeric waste is one of the most common plastics about 70% of total plastic bottles consumption (Meawad and Ibrahim 2019). Since PETs packaging materials wastes are not biodegradable, it will persist in nature for centuries of years. The European Packaging and packaging waste directive 94/62/EC (which deals with the problems of packaging waste) obligates member states to meet targets for the recovery and recycling of packaging waste (Rubio et al. 2019).

Plastic waste is considered one vital challenge beside environmental water contamination thorough nuclear and/or industrial activities. These activities are responsible for releasing toxic as well as radioactive elements into the water bodies (Cotruvo 2017; Bain et al. 2020). Uranium is one of the most harmful elements that threaten human health and the ecological environment due to its radioactivity and carcinogenicity. The maximum uranium contamination limit had reported by the U.S. Environmental Protection Agency to be 15 pCi L^−1^ (Al Nuaimi and Williams 2022). Uranium mining and milling, radioactive industries, and waste disposal are examples of human activities that contaminated the aqueous environment with uranium (Hussein and Taha 2013; Al Nuaimi and Williams 2022).

Uranium removal from contaminated water is attracting more global attention for more environmental protection. Worldwide, several approaches have been developed for uranium elimination from aqueous solutions. These approaches include precipitation (Al Nuaimi and Williams 2022) (Mousa et al. 2013), ion-exchange resins (Khawassek et al. 2018) (Masoud 2020), nanofiltration (Khawassek et al. 2018; Masoud 2020), solvent extraction (Ali et al. 2014), and adsorption (Younes et al. 2018; Hassanein et al. 2021; Aslani and Amik 2021; Yakout et al. 2013; Kütahyali and Eral 2004). One of the most effective and promising methods for uranium removal from wastewater is the sorption technique owing to its eco-friendly, simplicity, and feasibility (Yakout et al. 2013; Kütahyali and Eral 2004).

Numerous materials have been applied and optimized for the removal of uranium from an aqueous solution such as clays (Taha et al. 2018), bio-chars (Jin et al. 2018), zeolites (Ghaly et al. 2018), polymers (Gamal 2021), and activated carbons (Aslani and Amik 2021; Yakout et al. 2013; Kütahyali and Eral 2004; Belgacem et al. 2014). One of the most common sorbents for wastewater treatment is activated carbons (ACs). Activated carbons have been applied widely for uranium sorption from radioactive liquid effluents (Kütahyali and Eral 2004; Belgacem et al. 2014). Aslani and Amik produced AC/PAN (active carbon/polyacrylonitrile) composite as a steady and high capacity sorbent for U(VI) separation from wastewater (Aslani and Amik 2021). Younes et al. investigated uranium(VI) sorption from aqueous solution using rice straw activated carbon modified with KOH (Younes et al. 2018). Kütahyalı and Eral applied chemical activated carbon for selective removal of uranium(VI) from aqueous solution (Kütahyali and Eral 2004). Belgacem et al. used the activated carbon converted from the waste tire for U(VI) removal from an aqueous solution (Belgacem et al. 2014). Gamal investigated commercial activated carbon for uranium sorption from acidic aqueous solution (Gamal 2021). Li et al. reported that the modified bio-char fibers generated from luffa sponges are a promising sorbent for the uranium removal process (Liatsou et al. 2017). Tian et al. developed oxime-grafted ordered mesoporous carbon for effective removal of uranium from aqueous solution (Tian et al. 2011).

Recycling of waste plastic through catalyzed pyrolysis has been studied over recent decades (Armenise et al. 2021). Complete decomposition in pyrolysis of waste plastics can be carried out over 450 °C to produce gas and liquid and solid products (de Marco et al. 2009). PET was used to prepared series of materials based on various applications depending on results of pyrolysis (Sogancioglu et al. 2020). ACSs were synthesized by activation of nickel-doped polymer beads to use in loading of VB12 for refinement and separation applications **(**Saraswat et al. 2012). Most common cases of precursors for such polymeric packaging materials adsorbents are methyl acrylate, styrene, and phenol (Nanda and Berruti 2021).

In this contribution, the present study is concerned with investigating the uranium(VI) elimination from aqueous solution using two kinds of active carbon-based waste packaging materials prepared at different pyrolysis temperature by batch kinetics and equilibrium studies.

## Experimental

### Materials

In this work, the utilized chemicals, namely nitric, sulfuric, and hydrochloric acid, were reagent grade (Adwic, Egypt). UO_2_(NO_3_)_2_·6H_2_O (99.9%, Aldrich, USA) was used for the preparation of U(VI) stock solution (1000 mg L^−1^). Milli-Q water was used as diluent. NaOH and/or HCl solutions (0.1 M) were used to control the pH of the prepared solution. PET plastic packaging bottles were collected from households domestic and from Messes disposables then crushed to define its diameters.

### Measurements and analysis

The particle size and zeta potential of active carbons based on waste polymeric packaging materials were analyzed using NICOMP 380 ZLS, PSS, Santa Barbara, CA, USA). N_2_ ads./des. was measured by NovaTouchLX4 Quantachrome, USA to calculate surface areas of active carbons prepared from waste polymeric packaging materials. Thermal analysis for the studied samples was measured using DSC131 evo, SETARAM Inc., France. The test was computerized with heating range from 25 to 400 °C with a rising temperature rate 10 °C/min. All measured samples were processed using (CALISTO Data processing software v.149). The morphology of active carbons was investigated by scanning electron microscopy (SEM) (Quanta FEG 250, USA). The FEI Quanta 250 FEG-SEM is equipped with schottky field emission gun and Everhart–thornley detector for (secondary electrons) to deliver ultrahigh resolution (1.2 nm @ 30 kV). Inductively coupled plasma optical emission spectrometer model Optima 2100DV (Perkin-Elmer, USA) was used for U(VI) measurement as well as chemical analysis for the waste working sample. Orion pH/mv 910 ion analyzer with accuracy =  ± 0.01 was applied for measuring solution pH. Thermostatic shaking water bath model G.F.L 1083, Germany (working temperature range of 25–100 °C) was applied for performing the batch experiments. Solid/liquid separation was achieved using Z-230 type centrifuge (Hermle, Germany) with 5500.0 ± 5.0 rpm maximum speed.

### Sorbent preparation

Simple and easy technique was selected to be applied in industrial scale with economic cost. The pyrolysis processes were carried out under stream of N_2_ gas in a horizontal tube furnace. In a usual experiment, 50 g of PET flakes is charged into the tube furnace. Then, N_2_ gas is passed with 0.9 dm^3^/min and the temperature is raised at 450 °C for (sample labeled as: A) and 500 °C for (sample labeled as: B) for 35 min using heating rate of 15 °C/min. Solid yields from pyrolysis were recorded by weighting the product and computing the related percentage for ACs, A (10.1 wt%) and B (9.7 wt%). The results presented in this work are the average values of the recorded data for at least three equivalent pyrolysis yields with no more than 0.2% difference.

### Experimental procedures

Uranium (VI) sorption experiments were performed batch-wise in polypropylene tubes using activated carbon sorbents under the ambient parameters. Unless otherwise stated, the liquid phase containing 100 mg uranium L^−1^ contacted with the solid sorbent at room temperature (298 K), sorbent dose (SD) of 3.0 g L^−1^, and solution pH of 3.99 for 12 h (to ensure that the equilibrium is reached). The sorption tests were performed in triplicate and 4% relative errors mean values were applied. Uranium (VI) concentration was measured using ICP-AES, as mentioned before, in the liquid phase after the disengagement of the two phases. Based on the displayed data, Eqs. 1–3 were applied for evaluating U(VI) sorption efficiency (R%), sorption capacity (qe, mg g^−1^), and the distribution constant (K_d_) respectively.

1 $$\mathrm{R \%}=\frac{\left({\mathrm{C}}_{\mathrm{o}}-{\mathrm{C}}_{\mathrm{e}}\right)}{{\mathrm{C}}_{\mathrm{o}}}\mathrm{X}100$$

2 $${\mathrm{q}}_{\mathrm{e}}=\left({\mathrm{C}}_{\mathrm{o}}-{\mathrm{C}}_{\mathrm{e}}\right)\mathrm{X}\frac{\mathrm{V}}{\mathrm{m}}$$

3 $${\mathrm{K}}_{\mathrm{d}}=\frac{\left({\mathrm{C}}_{\mathrm{o}}-{\mathrm{C}}_{\mathrm{e}}\right)}{{C}_{e}}\mathrm{X}\frac{\mathrm{V}}{\mathrm{m}}$$

where the U(VI) concentration at the beginning (Co, mg g^−1^) and the end (Ce, mg g^−1^) of the sorption process, the volume of the aqueous phase (V, L), the weight of the sorbents (m, g), and the sorption capacity is (qe, mg g^−1^).

## Results and discussion

**Fig. 1:**
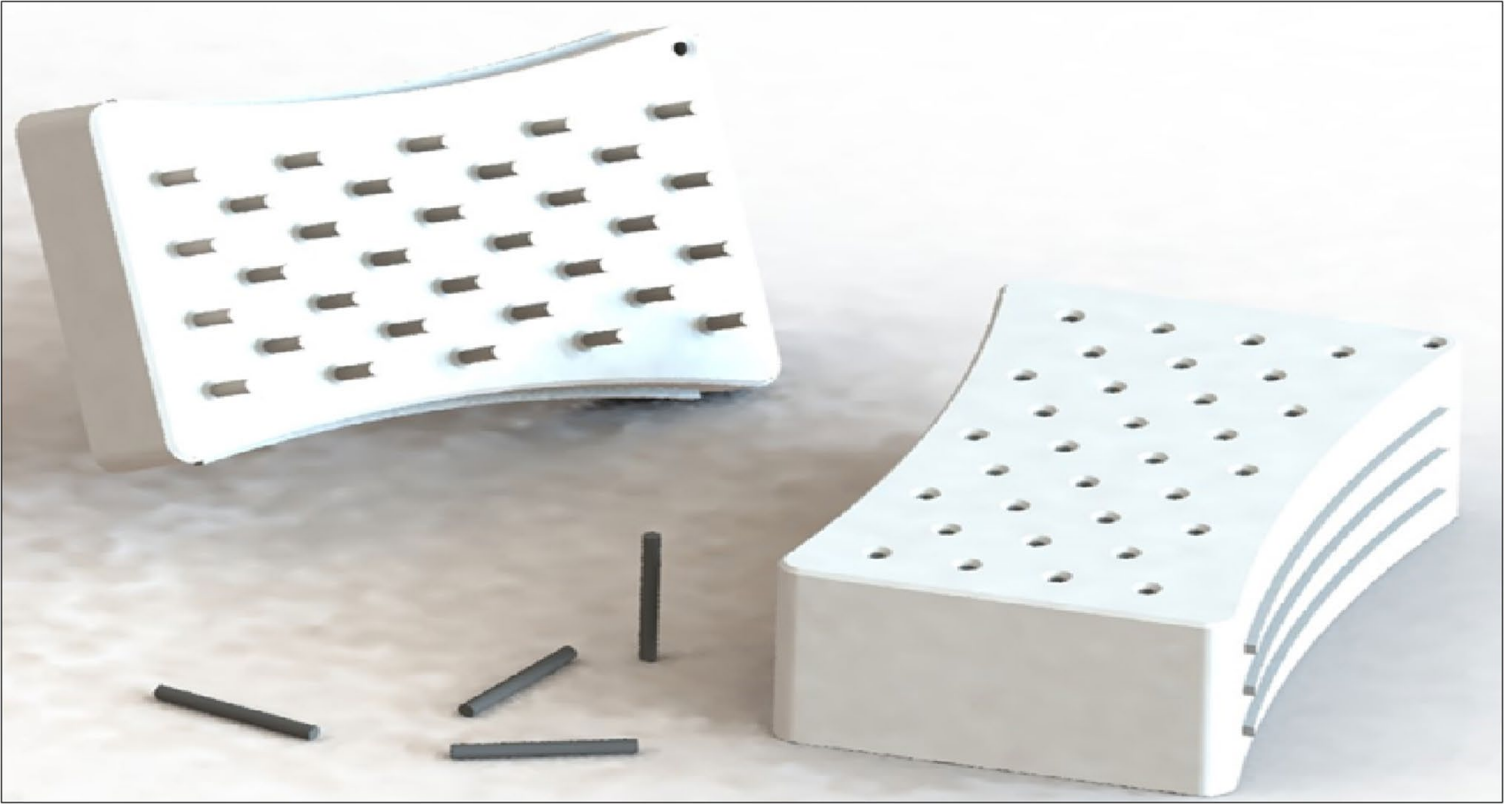
The mold designed to compress the prepared active carbon powder-based from recycling plastic packaging materials

The mold form was designed through solid Works program 2019 with dimension 80 mL × 130 mL × 42 mL with positive cylinder nips 10 mL (Fig. 1). The mold was printed with 3D printing technology using acrylonitrile butadiene polymer (ABS) having good mechanical properties and thermal stable with excellent abrasion resistance. Mold was designed within two parts, negative molds consist of a set of cylindrical cavities in which recycled plastic packaging materials as active carbons powder were charged. In addition, positive part was used as manual pressing to comprise the powder into cylinder shape. Ribs were applied to increase the mold strength during compression process of carbon powder. Mold side walls were modified with real texture, bumps appearing, for easy control during pressing process. Moreover, this extrusion is suitable for anatomy of hands and to support the structural design and prevent slipping.

### Sorbent characterization

Zeta potential measurements as an overall charged were used as display to the dispersion stability of active carbon in solutions (Lu et al. 2018). Prepared AC samples were dispersed with constant concentration under sonication condition. The zeta was investigated as an average to multi-measurements under low voltage. The average frequency shift and mobility of AC particles were indicated to a low packing effect by charge/size mobility particles (Ibrahim et al. 2019).

The average zeta potential measurements of active carbons prepared from waste polymeric packaging materials before and after the treatments are collected in Table 1. The zeta potentials of the two types of active carbon before treatments Ab and Bb are − 7.17 and − 25.63 mV, respectively, which indicated to moderate emulsion stability. Negative charge of investigated ACs will be enhanced the extraction ability of uranium as will be discussed later.

**Table 1:** Average zeta potential and relative indices of active carbons prepared from waste polymeric packaging materials before and after sorption process

Sample	Cell current, mA	Frequency shift, Hz	Avg. mobility, M. U	Avg. zeta potential, mV
Ab (before)	0.72	− 0.31	− 0.34	− 7.17
Aa (after)	1.06	− 0.46	− 0.50	− 4.86
Bb (before)	0.83	− 2.00	− 1.79	− 25.63
Ba (after)	1.16	− 0.74	− 0.74	− 10.55

**Fig. 2:**
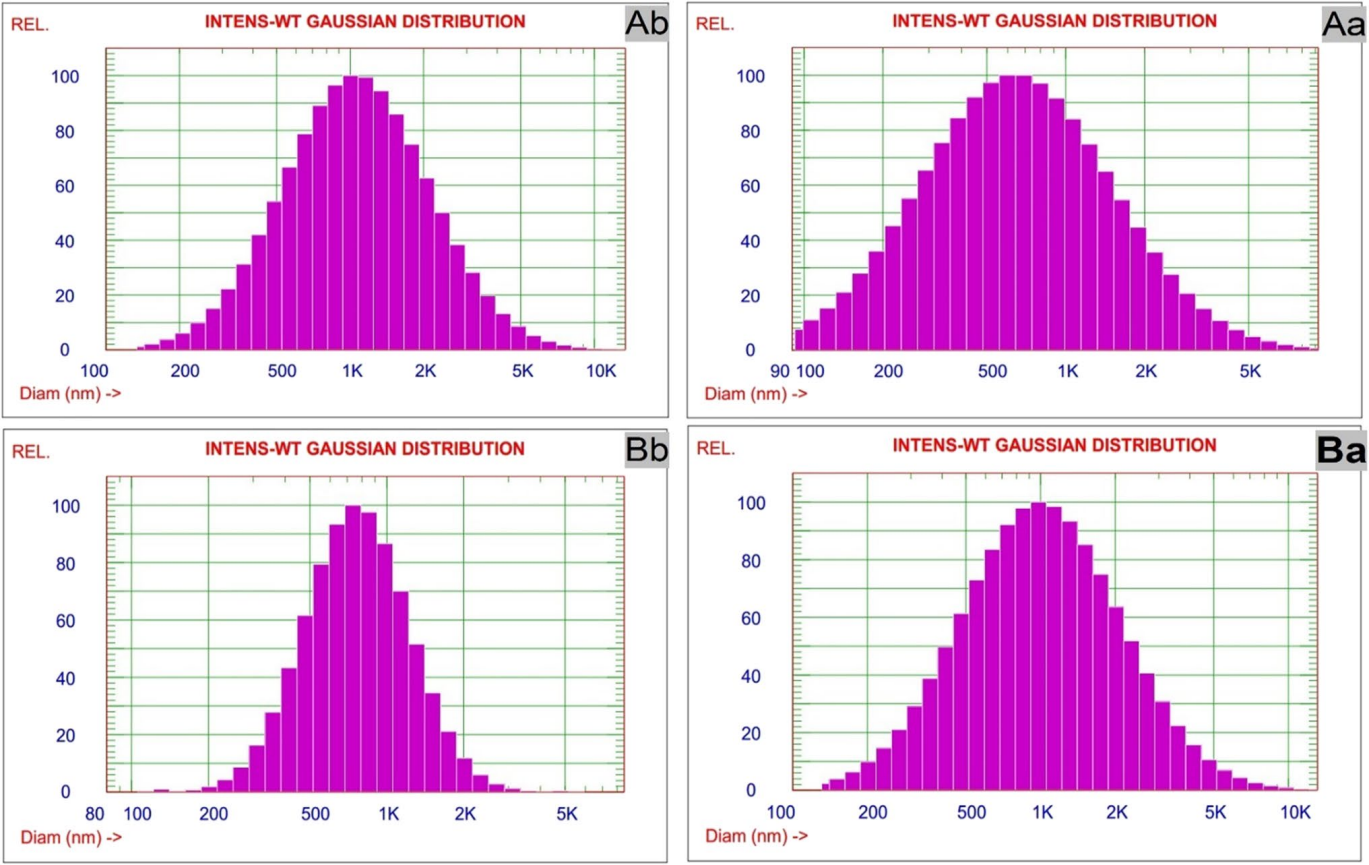
The particle size distribution of active carbons prepared from waste polymeric packaging materials (Ab, carbon A before; Aa, carbon A after; Bb, carbon B before; Ba, carbon B after)

The particle size intensity-weighted Gaussian distributions of active carbons prepared from waste polymeric packaging materials for two types of AC before and after treatment are shown in Fig. 2. Uni-modal (bell shape) homogeneous distributions of particle size were exhibited for ACs as indication to uniform distribution (Ibrahim et al. 2019). The particle size can be affected by the surface adsorption that varying the shape, density, and surface charge (Ibrahim and Sultan 2019).

The mean diameters of these dispersed active carbons prepared from waste polymeric packaging materials are tabulated in Table 2. The particle size was decreased in case of AC (Aa) was lower than related AC (AB). Additionally, the mean diameter was reduced by 31% in case of active carbon type B. These results can be attributed to the adsorbate which enhanced the dispersion of ACs.

**Table 2:** The hydrodynamic diameters and polydispersities of active carbons prepared from waste polymeric packaging materials

Sample	Mean diameter (nm)	Stnd. dev. (nm)	Polydispersity (PDI)	Chi square
Ab	817	751.4	0.84	105.5
Aa	779	514.8	0.44	143.0
Bb	1074	1021.8	0.90	254.4
Ba	733	497.5	0.46	137.8

**Fig. 3:**
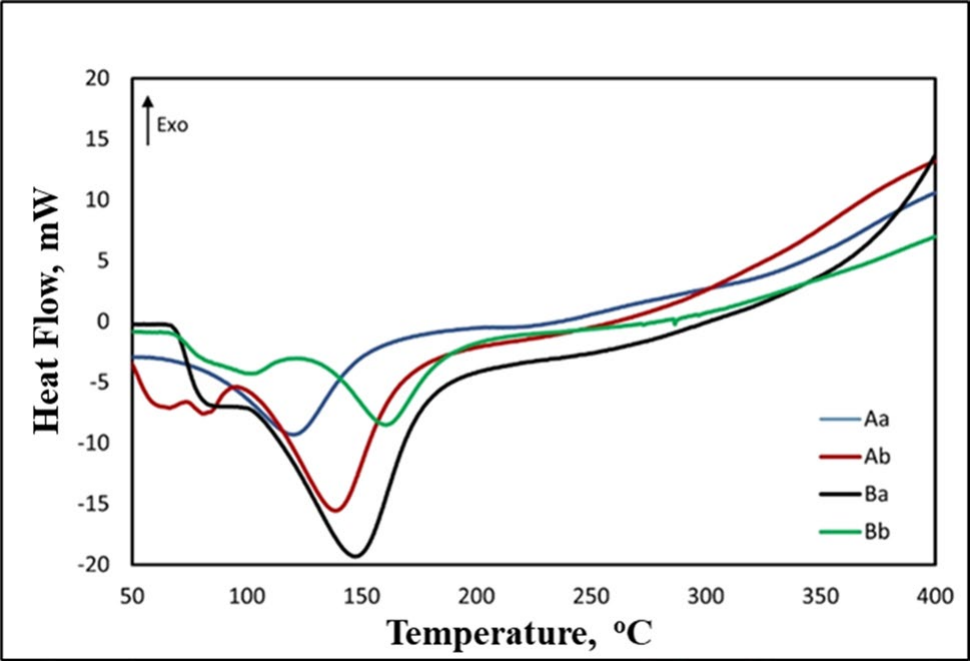
DSC thermograms of active carbons prepared from waste polymeric packaging materials before and after treatment (Ab, carbon A before; Aa, carbon A after; Bb, carbon B before; Ba, carbon B after)

DSC was used as an effective and high precision heat gain instrument to investigate the thermal behaviors of active carbons prepared from waste polymeric packaging materials. Figure 3 exhibited thermograms in the range of (0–400 °C) of the prepared ACs before and after treatment.

Active carbons prepared from waste polymeric packaging materials were presented smooth thermographs with main characteristic peak in the range of 120–148 °C (Zhou et al. 2008; Lee et al. 1975). According to DSC thermograms measurements, the onset temperatures (T_o_) of active carbons prepared from waste polymeric packaging materials Ab, Aa, Bb, and Ba are 92.7, 86.3, 95.9, and 67.9 °C, respectively. Additionally, the offset temperatures (T_f_) of Ab, Aa, Bb, and Ba are 148.3, 146.7, 143.2, and 132.9 °C with maximum temperature (T_m_) 124.0, 120.4, 119.1, and 105.9, respectively. On the other hand, the heat enthalpy of the samples was presented little difference between after and before treatment by 37% for sample A and 3% for sample B.

The peak maximum shifts can be handled according to two main assumptions: first, the ability of active carbons to gain heat after treatment early than raw samples. The second assumption, the addition of metal form on active carbon surface through adsorption as physical composite’s structure was enhanced the phase interaction and improved the compatibility of metal form ACs/U composites phases which reflected on the left shift of peak maximum temperatures.

The surface parameters of the active carbons prepared from waste polymeric packaging materials are tabulated in Table 3. The surface parameters of the prepared active carbons-based waste polymeric packaging materials were investigated to confirm the area/volume relationship.

**Table 3:** Surface parameters of the active carbons prepared from waste polymeric packaging materials

Sample	Total pore volume, cc/g	Average pore size, nm	Correlation coeff. (R)	Surface area (cm^2^/g)
Ab	1.357 e-001	2.014	0.999	544.9
Aa	3.285 e-002	1.800	0.998	337.3
Bb	4.274 e-002	2.084	0.999	632
Ba	9.381 e-002	1.773	0.998	416.2

The surface area was decreased after treatment for ACs A and B as shown in Table 3. This is due to the packaging effect of the adsorbed metal particles which encourages the aggregation effect (Lee et al. 1975). The total pore volume (TPV) and average pore size (APs) are tabulated in Table 3 with relevant decrease of TPV and APS after treatment than before. Moreover, the correlation coefficient is nearly ≈1 as first order relationship. Although there is a decrease in the surface area, however, the results are in agreement with AC/metal composites structures. Overall, the surface area measurements were agreed with particle size investigation as shown in Tables 2 and 3.

**Fig. 4:**
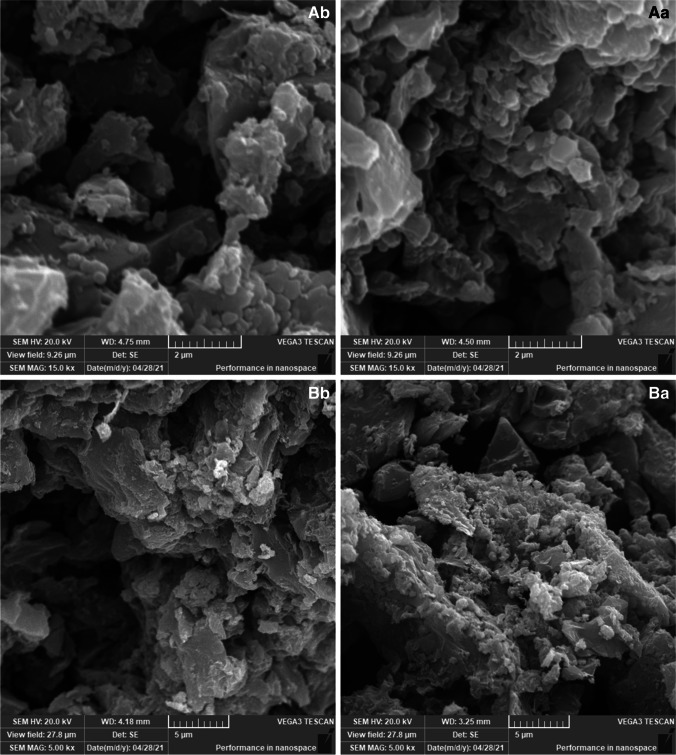
SEM images of the active carbons prepared from waste polymeric packaging materials before (Ab and Bb) and after treatment (Aa and Ba)

SEM was used to investigate the properties of the active carbons prepared from waste polymeric packaging materials before and after treatment. The images with high magnification were presented in comparing between active carbon before and after treatments as shown in Fig. 4. The space between active carbons as rocks form was clear in sample Ab and Bb. On contrast, the hole among the active carbons was closed by adsorbed materials.

### Batch experiments

The following section represents the sorption performance of carbon A and B. The main factors influencing the sorption process have been investigated, batch-wise, to achieve an effective removal of U(VI) from the aqueous solution. The investigated parameters are solution pH (1–7), shaking time (2–720 min), reaction temperature (25–50 °C), metal ion initial concentration (20–300 mg L^−1^), and sorbent dose (0.3–5 g/L).

#### Effect of pH

**Fig. 5:**
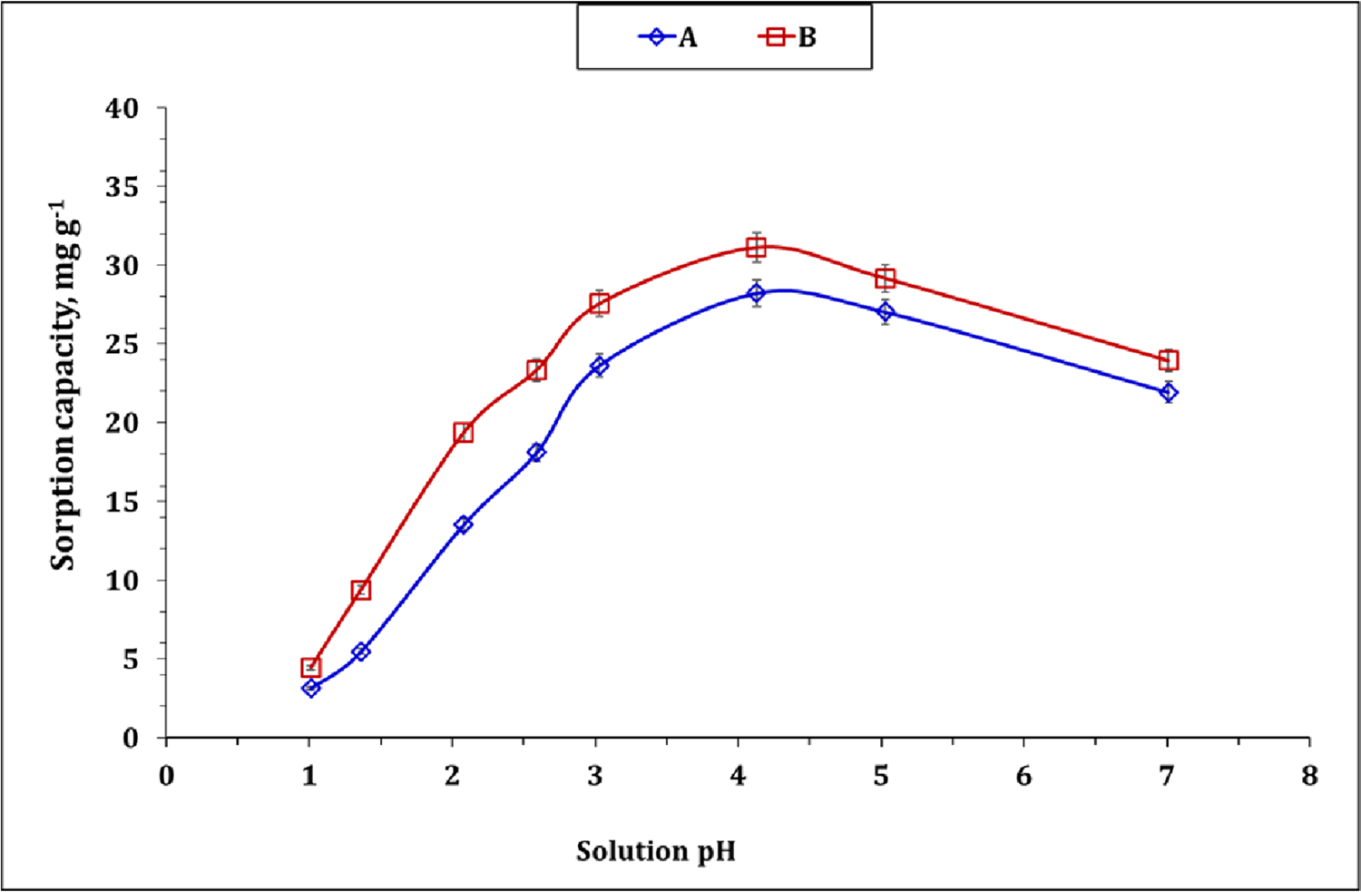
U(VI) sorption capacity as a function of solution pH (mixing time: 12 h; room temperature; U(VI) initial concentration: 100 mg L.^−1^; and sorbent dose: 3 g/ L)

The sorption performance of carbon A and B has been studied as a function of solution pH ranging from 1 to 7. Other variables were 3 g/L sorbent dose, U(VI) initial concentration of 100 mg L^−1^, room temperature, and mixing time of 12 h. The displayed data in Fig. 5 shows that solution pH has a positive impact on the sorption characteristics of both sorbents up to pH 4.0. The maximum sorption capacity was about 28.2 and 31.1 mg g^−1^ for carbon A and B respectively. Further increase in solution pH accompanied by a gradual decrease in the sorption capacity (negative impact). The sorption behavior of both sorbents at different solution pH could be attributed to the variation of uranium speciation as well as the chemical properties of the sorbent surface as the solution pH varied (Younes et al. 2021; Elzoghby et al. 2021).

Figure S1 displays the speciation of uranium metal ion (100 mg L^−1^) as a function of solution pH using Medusa/hydra software (Hussein and Taha 2013). The explored data shows that uranium possesses cationic species (mainly UO_2_(NO_3_)^+^ and UO_2_^2+^) at pH up to 4.0, while uranium exhibits neutral species (mainly UO_2_(OH)_2_.H_2_O_(S)_) at a pH range of 4.0–12.0, and finally, the negative uranium species is explored mainly at pH ≥ 12.0. The same species have been reported previously in other works (Hussein and Taha 2013; Khawassek et al. 2018; Younes et al. 2018; Hassanein et al. 2021).

According to Table 1, the surfaces of carbon A and B carry a negative charge (− 7.17 and − 25.6 mV respectively) which means that it will have a tendency for attracting the uranium cationic species at pH up to 4.0. Moreover, the decrease in zeta potential of the applied sorbents before and after U(VI) sorption process (from − 7.17 to − 4.86 mV for carbon A; and from − 7.17 to − 4.86 mV for carbon B) confirms the accumulation of uranium cationic species on the sorbent surface. Briefly, in acidic solutions, the applied sorbents show low uranium sorption capacity. This performance could be attributed to the existence correct of high concentration of H^+^ ions which accumulates on the sorbents surface (Gamal 2021; Liatsou et al. 2017). As the solution pH increases, the concentration of H^+^ decreases which gives more chance for the interaction (mainely electrostatic interactions) between the sorbent’s surface and uranium(VI) species (Younes et al. 2021; Elzoghby et al. 2021). The decrease in U(VI) sorption capacity at higher solution pH is attributed to the disappearance of uranium cationic species and the presence of the insoluble species (UO_2_(OH)_2_.H_2_O_(S)_). The same sorption performance has been reported for U(VI) sorption from aqueous solution using active carbon/PAN composite (Aslani and Amik 2021), activated carbon from wood wastes (Alahabadi et al. 2020), and *phosphorylated luffa rattan activated carbon* (Zhang et al. 2021).

#### Sorption kinetic

**Fig. 6:**
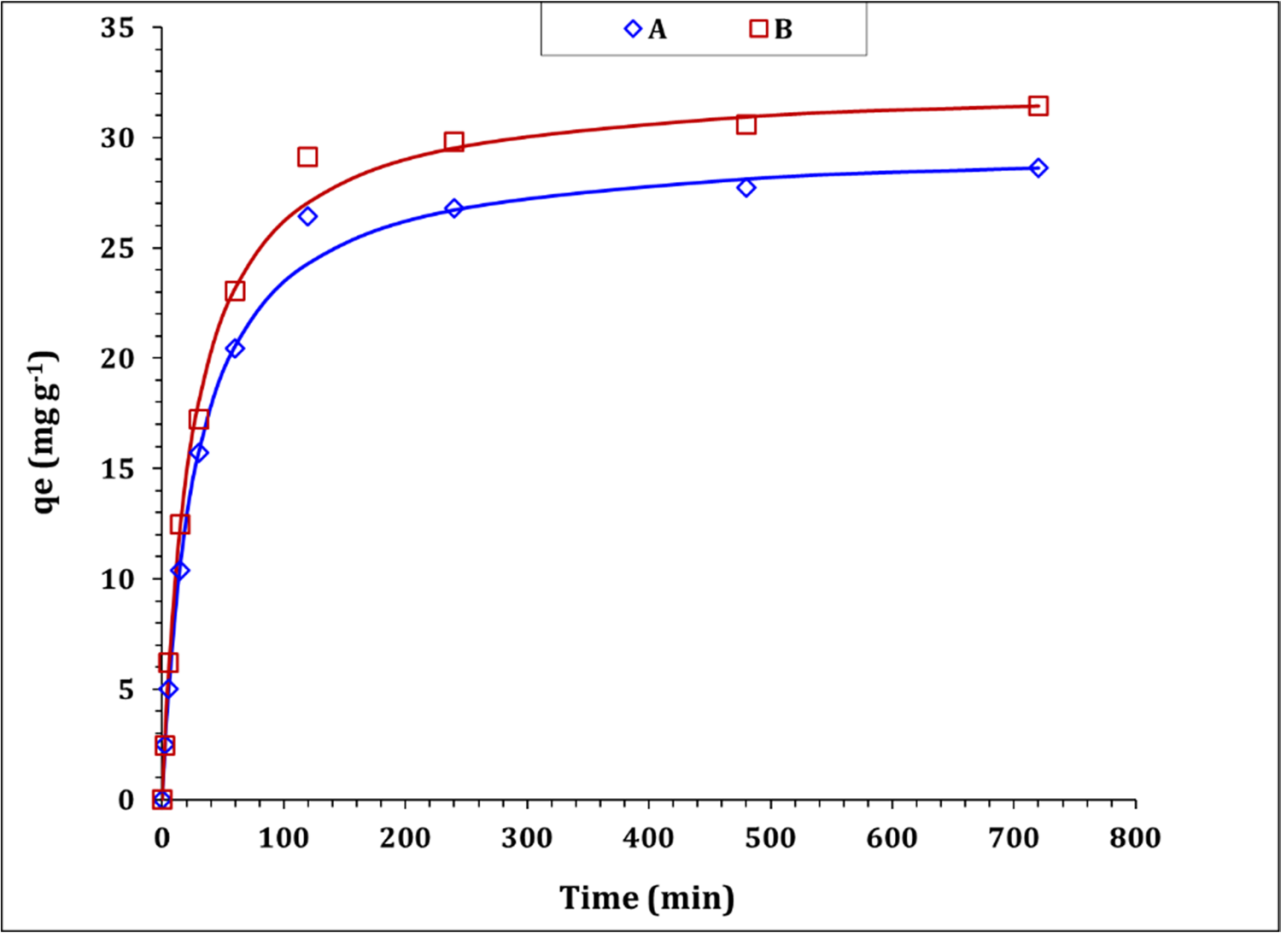
The kinetic curve of for U(VI) sorption using carbon A and B

The kinetics of U(VI) sorption from aqueous solution using carbon A and B were investigated to figure out the reaction rate-controlling step and discover the possible sorption mechanism. Accordingly, a group of tests has been conducted at a different time interval (2–720 min), while other parameters were fixed at solution pH of 3.99, sorbent dose of 3.0 g/ L, U(VI) initial concentration of 100 mg L^−1^, and the temperature of 25 ± 1 °C. The variation of sorption capacity (qe) as a function of mixing time (kinetic curve) was displayed in Fig. 6. From the figure, it is obvious that both sorbents exhibit the same kinetic profile, which is consisted of two phases: phase (I) fast rate of reaction which extends to about 120 min (the equilibrium time) and characterized by a rapid increase in sorption capacity. Phase (II) exhibits a much slower rate of reaction, whereas a steady-state of uranium sorption capacity could be observed. The sorption performance at phase (I) could be explained by the high availability of surface active sites and the high concentration gradient between the surface sorption sites and the solution which leads to a fast accumulation of U(VI) species on the sorbent surface (Tian et al. 2011). However, the behavior at the second phase is attributed to the saturation of the sorbent surface active sites and the resistance to intraparticle diffusion which requires a much longer time (Younes et al. 2021). About 80% and 90% of U(VI) were removed using carobon A and B respectively at equilibrium time.

The kinetic characteristics of the U(VI) sorption process could be declared by applying two kinetic models, namely pseudo-first-order (PFORE) and pseudo-second-order (PSORE) models to fit the collected data from Fig. 6. The non-linear forms of the applied models are displayed in Table S1. The validity of each model is checked by the coordination coefficient (*R*^*2*^) and the non-linear regression chi-square (*X*^*2*^), whereas the highest (*R*^*2*^) and the lowest (*X*^*2*^) values reflect the validity of the model (Marques et al. 2019; Kang and Kim 2019). The equations of the coordination and the chi-square coefficients are presented in Table S1 (Marques et al. 2019; Kang and Kim 2019). The values of the kinetic parameters as well as the coordination and the chi-square coefficients are reported in Table 4. The explored data obvious that PFORE and PSORE kinetic models exhibit perfect correlation coefficient value (0.99) for both applied sorbents, while PSORE displays the lowest chi-square coefficient (0.3) for carbon A and B. In addition, the calculated qe values for the applied sorbents at equilibrium (29.7 mg g^−1^ for A; 32.5 mg g^−1^ for B) are consistent with the experimental qe values (28.6 mg g^−1^ for A; 31.4 mg g^−1^ for B) which confirms that PSORE kinetic model is more appropriate to describe the process of uranium(VI) sorption using both martials. This indicates that the rate-controlling step is chemisorption, and the sorption process might involve electron sharing and/or transfer between the metal ions and the active functional groups on the applied sorbents (Younes et al. 2021; Elzoghby et al. 2021). The same kinetic profile has been reported for uranium(VI) sorption from aqueous solution using active carbon/PAN composite (Aslani and Amik 2021), activated carbon from wood wastes (Alahabadi et al. 2020), and *phosphorylated luffa rattan activated carbon* (Zhang et al. 2021).

**Table 4:**
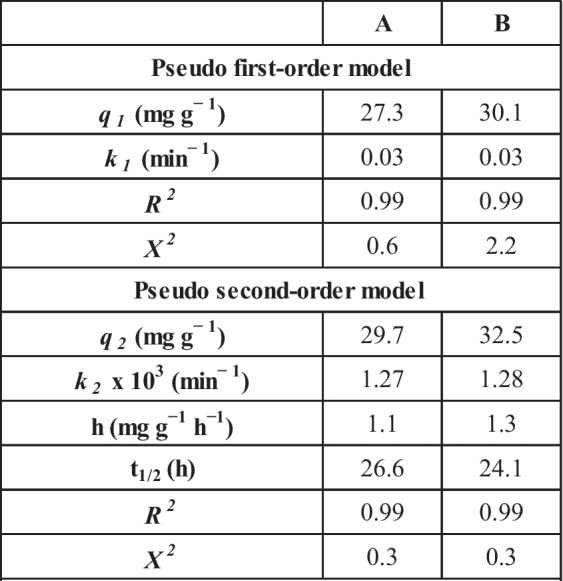
The evaluated kinetic parameters; pseudo first order, and pseudo second order models

Table 4 obvious that the half equilibrium time (t_1/2_) and initial sorption rate for carbon A are 26.6 h and 1.1 mg g^−1^ h^−1^ respectively, while the same terms for carbon B are 24.1 h and 1.3 mg g^−1^ h^−1^ respectively. In addition, sorbent B exhibits higher uranium sorption capacity than sorbent A at equilibrium. This reflects that carbon A exhibits better sorption characteristics than carbon B. This characteristic could be owing to the variation in sorbent surface area (Table 1), whereas sorbent B possesses a higher surface area (632.1 m^2^/g) than sorbent A (544.9 m^2^/g), meaning more active functional groups for U(VI) binder. In addition, the surface negative charge of carbon B (− 25.6 mV) is higher than that of carbon A (− 7.17 mV) which reflects a higher tendency for the attraction of the cationic uranium species (Jin et al. 2018).

**Fig. 7:**
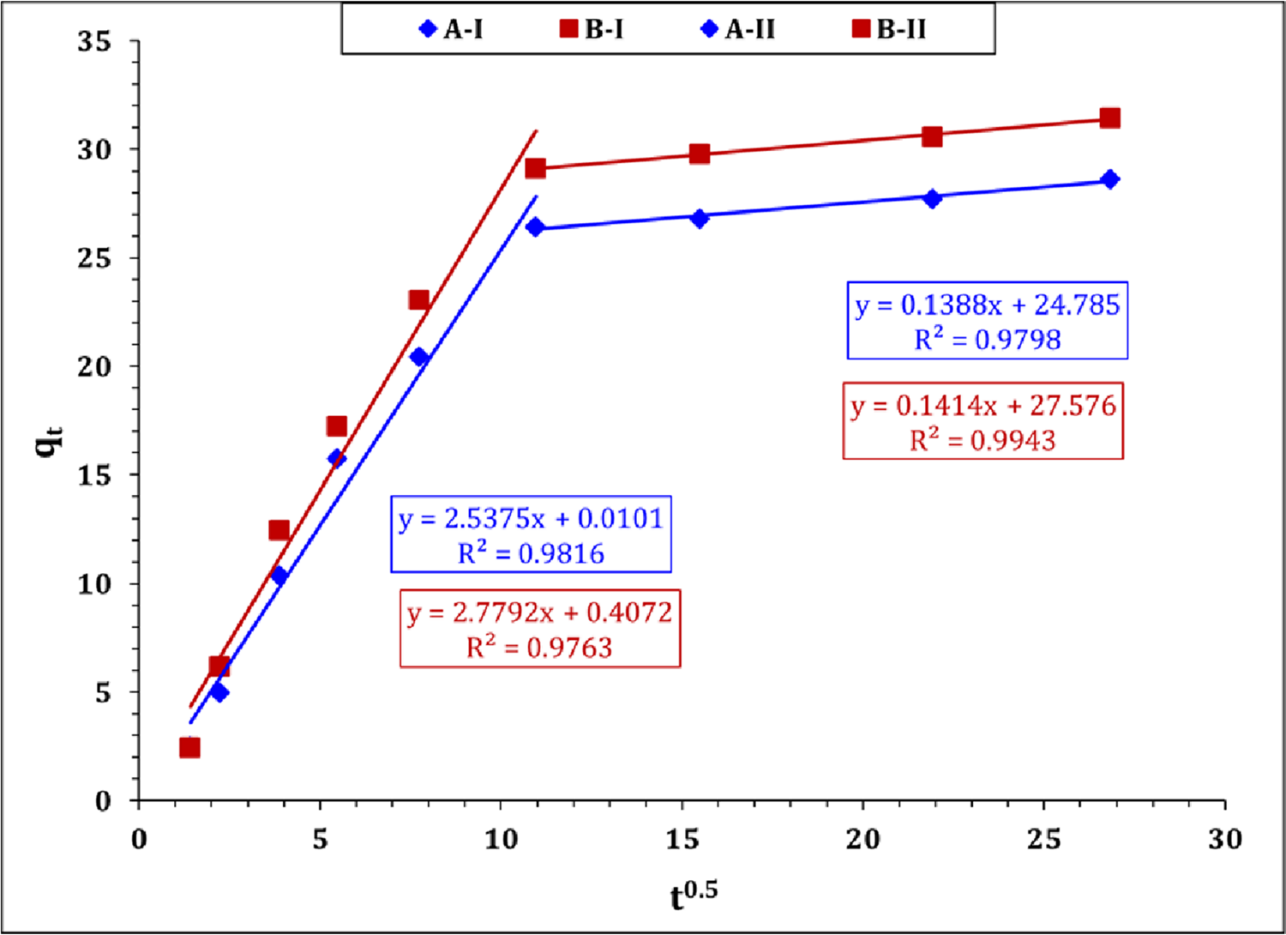
Morris–Weber model plot for U(VI) sorption using carbon A and B

Intraparticular diffusion model (IPD) which also known as Weber–Morris kinetic model was applied to investigate the impact of resistance to intra-particle diffusion, and provide a more comprehensive description for the sorption mechanism (Hussein and Taha 2013; Ahmed et al. 2020). The linear form of the Morris − Weber model is displayed in Table S1, while the model parameters, namely C and ki, were evaluated and shown in Table S2. The variation of sorption capacity qt as a function of t0.5 (Weber − Morris plot) is displayed in Fig. 7. The plot displays two linear segments (multi-linear relationship) which indicates that U(VI) sorption using carbon A and B is controlled by multi-mechanisms. (Marques et al. 2019; Kang and Kim 2019; El-Korashy et al. 2016).

From the figure, it is clear that the sorption process includes two steps: step (I) is characterized by rapid sorption rate of reaction until the equilibrium (fast kinetics), which could be due to the availability of free surface active sites, so the reaction rate and/or resistance to film diffusion control the sorption mechanism (El-Korashy et al. 2016). Step (II) is characterized by a pseudo saturation plateau (slow kinetics) which might be attributed to the saturation of sorbent surface active sites, so the resistance to intraparticle diffusion takes place (El-Korashy et al. 2016). The results in Table S2 declare that the first stage exhibits a low boundary layer effect and a high rate of reaction (fast rate of reaction), while the second stage shows a high boundary layer effect and low rate of reaction (slow rate of reaction). Uranium (VI) sorptions from aqueous solution using activated carbon from wood wastes (Alahabadi et al. 2020), rice husk magnetic biochar composites (Li et al. 2019), and hydroxyapatite-biochar nanocomposite (Hamed et al. 2019) are also characterized by multiple mechanisms of sorption reaction.

#### Effect of the adsorbent amount

**Fig. 8:**
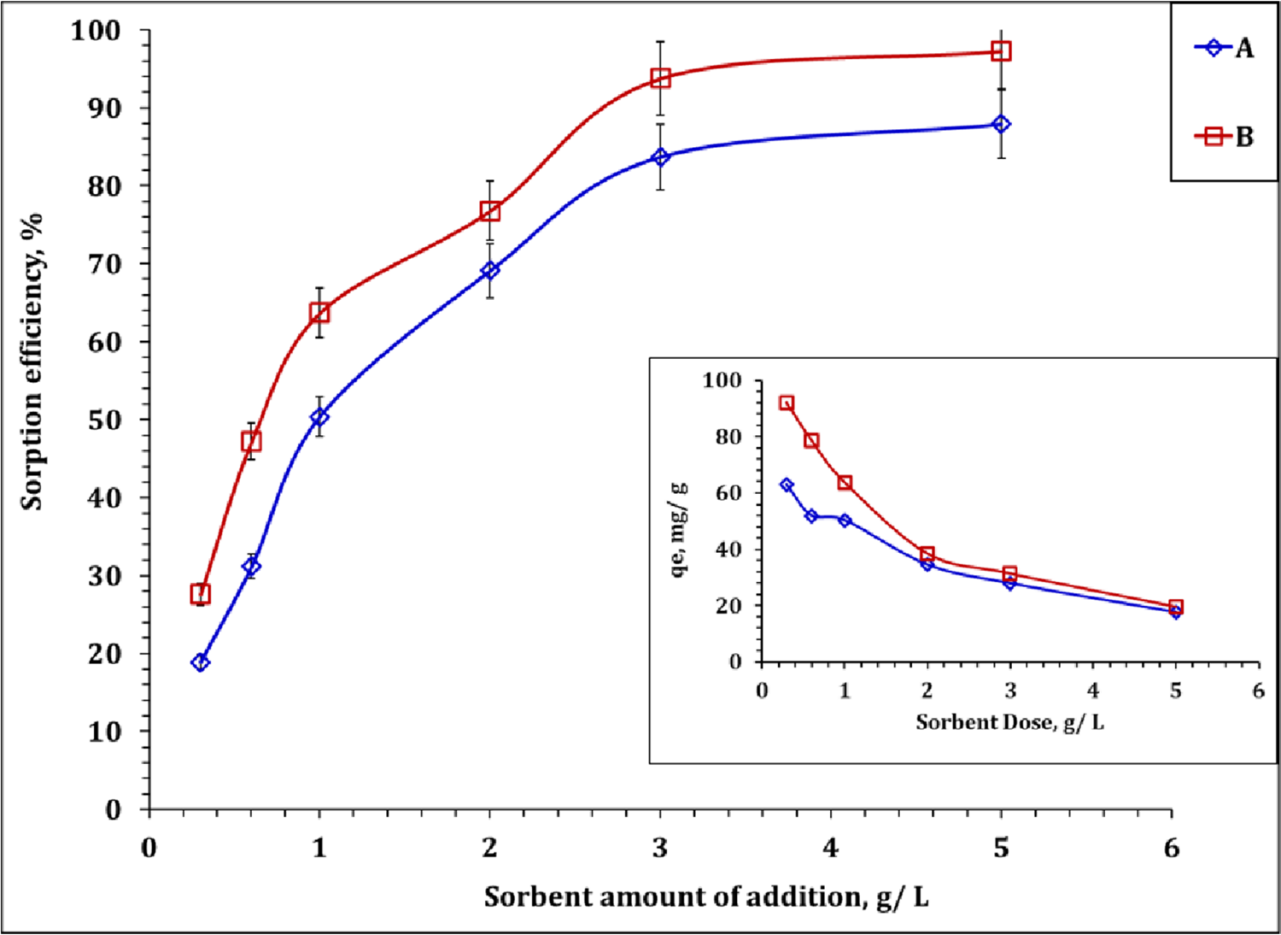
Uranium ions sorption efficiency and capacity as a function of sorbent dose

U(VI) sorption from aqueous solution using carbon A and B was investigated as a function of sorbent dose. Briefly, several experiments were carried out at a pH of 3.99, room temperature, U(VI) initial concentration of 100 mg L^−1^, and mixing time of 4 h; however, the sorbent dose changed from 0.3 to 5.0 g/L. The displayed data (Fig. 8) declares the positive variation of the U(VI) sorption efficiency for both sorbents as the sorbent increases up to 3.0 g/L, followed by a slight increase with the sorbent’s dose increase. This performance could be expanded as that at a low sorbent amount of addition, all sorbent’s active functional groups are available and interact with the U(VI) species. However, as the sorbent’s dose increases, the sorbent’s active sites become more excess than the existing U(VI) species; therefore, the surface saturation does not occur (El-Korashy et al. 2016). It is worth noting that the sorption capacity dramatically decreased as the applied sorbent dose increased. Selection of the preferred sorbent dosage depends mainly on the goals of the sorption process; maximum decontamination or concentration effect. In this regard, based on the collected data, 3.0 g/L represents a suitable dose for achieving about 94.0% removal efficiency (which is preferred for the environmental purposes) and in the same time, exhibits a suitable sorption capacity for the enrichment of U(VI) after sorption/desorption cycle.

#### Sorption isotherm

**Fig. 9:**
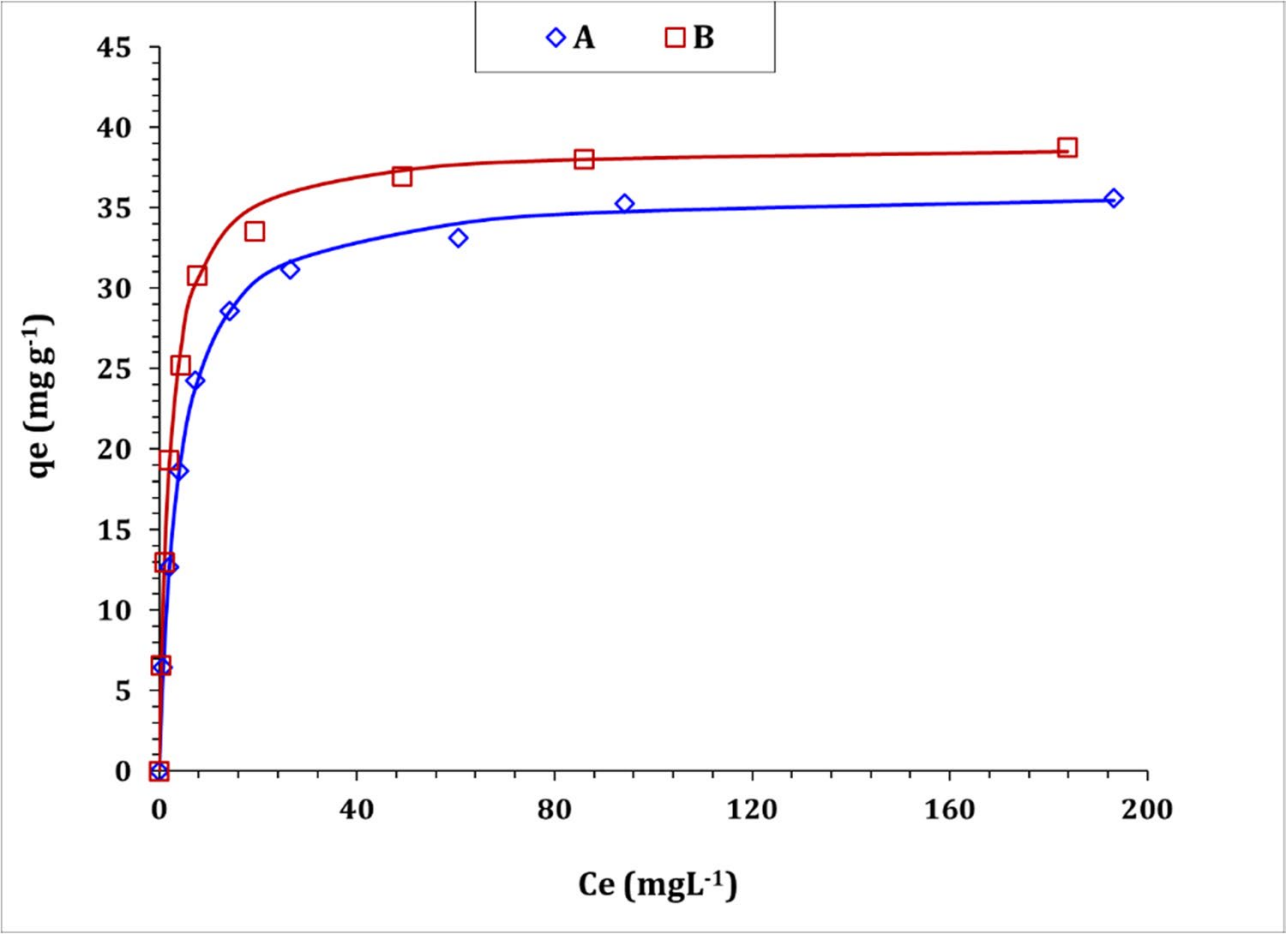
Isotherm profile for U(VI) sorption using carbon A and B

Uranium (VI) sorption isotherm (which reflects the distribution of U(VI) species between the applied sorbent and the aqueous solution at equilibrium) is a key parameter for improving the operational control as well as the plant design (Younes et al. 2018; Hassanein et al. 2021). Accordingly, several tests were conducted at U(VI) initial concentration interval (20–300 mg L^−1^); however, other variables were set at pH of 4.01, sorbent dose of 3.0 g/L, at 25 ± 1 °C for 4 h. The isotherm performance is presented by the variation of sorption capacity (qe) as a function of U(VI) residual concentration (Ce) (Fig. 9). The displayed results declare that at low U(VI) residual concentration, the sorption capacity of both sorbents is rapidly increased (first step), which reflects the presence of free active surface functional groups for interacting with U(VI) species (Younes et al. 2021)(Elzoghby et al. 2021). At the second step, the sorption capacity tends to be constant at high U(VI) residual concentration (plateau shape), which is attributed to the saturation of the functional groups on the sorbent surface (Younes et al. 2021; Elzoghby et al. 2021).

The isotherm profile of U(VI) sorption using carbon A and B could be explained using Langmuir, Freundlich, and Temkin, isotherm models. The non-linear form of the applied three isotherm models is shown in Table S1 (Marques et al. 2019) (Kang and Kim 2019). Both the coordination coefficient (*R*^*2*^) and the non-linear regression chi-square (*X*^*2*^) have been applied to test the validity of the isotherm models. The parameters of the isotherm models were evaluated and displayed in Table 5. The collected data in Table 5 shows that Langmuir model was fitting well the U(VI) sorption process using both sorbents, whereas it possesses the highest coordination coefficient (*R*^2^: 1.00), and the lowest chi-square coefficient (carbon A: 0.34 and carbon B: 0.13) which reveals that Langmuir model is more appropriate to describe the sorption process. The explored data in Table 5 shows that carbon B possesses a higher sorption capacity (qm: 39.3 mg g^−1^) than carbon A (qm: 36.6 mg g^−1^). The variation of the sorption capacity could be attributed to the variation in physical properties of both carbons whereas carbon B exhibits higher surface area and surface negative charge (632.1 m^2^/g and − 25.6 mV respectively) than that of carbon A (544.9 m^2^/g and − 7.17 mV) which means higher active surface functional groups and higher affinity to attract the positive uranium cations (Jin et al. 2018). Based on the displayed data, it could be assumed that monolayer adsorption occurs (the sorption occurs at equivalent active sites). Moreover, the sorption is chemisorption in nature, and the sorbent exhibits a homogenous surface which is in agreement with the SEM analysis (Fig. 4).

**Table 5:** The evaluated variables of Langmuir, Freundlich, and Temkin isotherm models

	A	B
Langmuir model		
q_m_ (mg g^−1^)	36.2	38.9
k_L_ (L mg^−1^)	0.26	0.47
*R*^*2*^	1.00	1.00
*X*^*2*^	0.34	0.13
Freundlich model		
1/n_F_	0.2	0.2
k_F_ (mg g^−1^) (mg L^−1^)	14.24	17.43
*R*^*2*^	0.87	0.83
*X*^*2*^	6.98	9.40
Temkin model		
b_T_ (J mol^−1^)	461.1	444.5
A_T_ (L g^−1^)	7.9	15.3
*R*^*2*^	0.94	0.92
*X*^*2*^	2.18	3.40

The same isotherm performance (monolayer and chemisorption sorption process) has been reported for U(VI) sorption from aqueous solution using active carbon/PAN composite (Belgacem et al. 2014), activated carbon from wood wastes (Alahabadi et al. 2020), and *phosphorylated luffa rattan activated carbon* (Zhang et al. 2021).

The displayed sorption capacity of carbon A and B were explored in comparison with the sorption capacity of other sorbents in Table 6. The explored data declares that the sorption capacity of carbon A and B is moderate in regard to the displayed sorbents. In addition, according to the low cost as well as the small dosage, the applied carbon sorbents are considered a potential material for cleaning contaminated aqueous solution from uranium species.

**Table 6:** Comparison of sorption performance of U(VI) for different carbon sorbents

Co, mg L^−1^	Temp, °C	pH	Time, h	Qe, mg g^−1^		Ref
10–400	30	4.0	2	28.2	Active carbon/polyacrylonitrile composite	**(**Aslani and Amik 2021**)**
50–200	30	5.0	3	28.4	Chemical activated carbon	**(**Kütahyali and Eral 2004**)**
2–60	45	4.0	24	52.6	Rice husk magnetic biochar composites	**(**Li et al. 2019**)**
20–300	25	5.5	1	27.2	Eucalyptus wood biochar	**(**Mishra et al. 2017**)**
5–100	20	4.0	6	19.4	Fungus Pleurotus ostreatus	**(**Zhao et al. 2016**)**
10–80	25	10.0	7	53.2	Modified rice husk biochar	**(**Wang et al. 2018**)**
10–110	25	6.0	1	62.7	Pine needles biochar by HTC	**(**Zhang et al. 2013**)**
20–240	30	6.0	2.5	16.3	Hazelnut shell activated carbon	**(**Zhu et al. 2016**)**
25–125	30	3.0	3.0	8.6	Pistacia vera L. shell activated carbon	**(**Donat and Erden 2017**)**
**20**–**300**	**25**	**4.0**	**4**	**36.6**	**Carbon A**	**Present work**
**20**–**300**	**25**	**4.0**	**4**	**39.3**	**Carbon B**	**Present work**

#### Sorption thermodynamics

Figure S2 represents the influence of temperature variation from 25 to 50 ± 1 °C on U(VI) sorption from aqueous solution using carbon A and B. The sorption conditions were fixed at pH of 4.02, sorbent dose of 3.0 g/L, U(VI) initial concentration of 100 mg L^−1^, and mixing time of 4 h. The displayed data explores that U(VI) sorption efficiency decreased with the increase of reaction temperature, which reflects the exothermic nature of the sorption process. Gibbs free energy change (ΔG°), standard enthalpy change (ΔH°), and standard entropy change (ΔS°) could be evaluated based on the following equations (Elzoghby et al. 2021):

4 $$\mathrm{log}{k}_{C}=-\frac{{\Delta H}^{o}}{2.303R}X\frac{1}{T}+C$$

5 $${-\Delta G}^{o}=2.303RT log{k}_{C}$$

6 $${-\Delta G}^{o}={\Delta H}^{o}-{T\Delta S}^{o}$$

where ${{\varvec{K}}}_{{\varvec{C}}}$ is a non-dimensional equilibrium constant $\left({{\varvec{K}}}_{{\varvec{C}}}={K}_{d} X \rho \times 1000\right)$ **[51]**; ${\varvec{T}}$ is the temperature (K), ***R*** is the universal gas constant (8.314 J mol^−1^.K^−1^), **ρ** is the solution density (g/ L), and ${\varvec{C}}$ is a constant.

The relation between log Kc *Vis* (1/T) (Van’t Hoff plot) could be used to determine the thermodynamic variables (Fig. S2). The explored results (Table 7) show that both sorbents exhibit negative ΔH° values (carbon A: − 21.8 and carbon B: − 56.9 kJ mol^−1^) which indicates the exothermic nature of the sorption process (Younes et al. 2021; Saha and Chowdhury 2011). The negative values of ΔG° for reaction temperatures range (25–50 ± 1 °C) for carbon A and B reflect the feasibility and spontaneous nature of the sorption process (Younes et al. 2018). Moreover, carbon A and B show negative ΔS° for carbon A and B (− 11.9 and − 119.3 J mol^−1^ K^−1^ respectively) indicating the decrease of randomness at the solid–liquid interface during the U(VI) sorption using the sorbent (Saleh et al. 2017; Metwally and Attallah 2019; Metwally et al. 2020). The same thermodynamic performance (i.e., exothermic, spontaneous, and feasible sorption process) has been reported for uranium ions sorption from aqueous solution using activated carbon developed from grinded used tire (Belgacem et al. 2014), and oxime-grafted ordered mesoporous carbon CMK-5 (Tian et al. 2011).

**Table 7:**
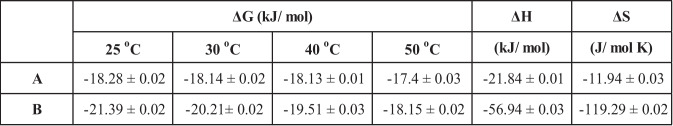
The evaluated thermodynamic parameters

#### Sorption mechanism

Identification of the uranium-sorbent interaction mechanism is very important for the development and assessment of remediation technologies. Uranium adsorption using activated carbon could involve numerous mechanisms, for example, complexation, electrostatic attraction, ion exchange, precipitation, and physical sorption (Xie et al. 2019; Kong et al. 2016). Uranium chemistry in the aqueous solution as well as the physical and chemical properties of the applied activated carbon sorbent mainly controlled the sorption mechanism (Yakout et al. 2013; Wang et al. 2020). Kong et al. investigated U(VI) sorption from aqueous solution onto nano-flake like Fe-loaded sludge carbon (Fe-SC). Based on the obtained data, both reductive precipitation and physical adsorption were the main mechanisms of immobilization of uranium from aqueous solution (Kong et al. 2016). Yakout et al. reported that U(VI) sorption from aqueous solution using rice straw activated carbon modified with KOH in the absence of humic acids could result from both ion exchange (replacing uranium mono cationic species [(UO_2_)_3_(OH)_5_^+^] with the proton from the carboxylic group of the carbon), and electron-donating acceptor complexation reactions between the de-localized π-electron of grapheme layers and uranium cationic species (Yakout et al. 2013). Wang et al. applied triethylenetetramine-functionalized single-walled carbon nanohorns as an efficient sorbent for U(VI) removal from aqueous solution, The displayed data shows that the sorption mechanism is mainly owed to the complexation between uranium species and the nitrogen-containing and oxygen-containing groups present on the sorbent surface (Wang et al. 2020).

The present work obvious that the Langmuir isotherm model is fitting well to the obtained results (Table 2) which means that the sorption process is chemisorption in nature and the interaction between uranium and the sorbent could be via electrostatic attraction mechanisms (Xie et al. 2019). Uranium is present as cationic species mainly UO_2_(NO_3_)^+^: ~ 66%, and UO_2_^2+^: ~ 34% (Fig. S1) at the studied pH interval (1.0–4.0), while both sorbents display a negative charge surface (carbon A: − 7.17 and carbon B: − 25.6 mV) at the same pH interval. In acidic solution pH, the solution is crowded with the H^+^ which means high competition with uranium cations for the attraction to the sorbent surface (low sorption efficiency). As the solution pH increase, the impact of H^+^ decreases, which give more probability to the electrostatic attraction between uranium cationic species and the negative sorbent surface (electrostatic attraction mechanism) (Yakout et al. 2013). According to the sorption kinetics, it is clear that U(VI) sorption process is obeyed to pseudo-second-order kinetic model (Table 1) which indicates that the sorption process is chemisorption in nature, and the π-interaction between U(VI) species and the surface function groups (i.e., γ-CH and C = C) might take place (Kang and Kim 2019). In addition, the variation in surface area as well the porous structure of the applied carbon (Tables 2 and 3) confirms that the physical sorption mechanism could contribute to the sorption process. According to the Morris–Weber plot (Fig. 7), it is clear that the uranium sorption process is controlled with multiple mechanisms and the interdiffusion mechanism is one of them. In this regard, uranium sorption using carbon a and b could be controlled mainly by electrostatic attraction mechanism at the beginning of the reaction, whereas there are enough sorbent surface active sites, while after equilibrium, most of the sorbent surface active sites becomes saturated; therefore, the intraparticle diffusion of U(VI) ions over the sorbent porous becomes the main sorption mechanism.

### Desorption and reusability investigation

The desorption potentiality and reuses are important parameters for sorbent feasibility. Accordingly, a set of experiments were carried out using (1.0 M) of the following mineral acids; nitric acid, hydrochloric acids, and sulfuric acid for U(VI) desorption from the loaded carbon B sorbent (which shows the highest sorption capacity). The elution experimental conditions were as follows: mixing time of 12 h, sorbent dosage of 3.0 g/L, and at room temperature. The efficiency of the U(VI) desorption process was evaluated using the change of U(VI) concentration before and after the elution process. The explored data in Table S3 displays that about 95.2% of the loaded U(VI) could be successfully desorbed from the loaded carbon B using 1.0 M sulfuric acid. Based on the previous data, the sorbent reuses were performed as a consequence of 5 sorption/desorption cycles. The revealed results in Fig. S3 declare that the sorption percent changed from 93.2 to 90.1%; however, the desorption percent changed from about 95.1 to 92.4% which indicates the high stability for the applied sorbents for a consequence 5 sorption/desorption cycles.

## Case study

Uranium removal from raffinate solution is an important process from the environmental point of view. Accordingly, carbon B sorbent undergoes an application experiment to eliminate uranium ions from a liquid waste solution produced at the Nuclear Materials Authority, Egypt. The main chemical compositions of the raffinate solution, as measured by ICP-AES, were as follows: Ca(II) and Fe(III) concentrations of 1.84 and 0.63 g L^−1^, nitric acid molarity of 1.0 M, and U(VI) concentration of 100 mg L^−1^. The experimental parameters were as follows: pH of 3.7, 3.0 g/L of carbon B, mixing time of 4 h, and room temperature. The collected data shows that about 90% of the uranium content in the raffinate sample was successfully eliminated, which reflects that the applied carbon sorbent is a good alternative material for the liquid waste remediation process.

## Conclusion

Active carbon-based waste polymeric packaging materials was successfully prepared through pyrolysis process at 450 and 500 °C. Prepared ACs were full characterized with unimodal distribution of particle size (817 and 1074 nm), zeta potential exhibit nice ACs dispersion (− 25.6 and − 7.17 mV), and surface areas (544 and 632 m^2^/g) were presented a good agreement with particle size. In addition, surface topographic was examined and compared before and after treatment for type A and B of ACs. Uranium removal from wastewater has been successfully achieved using both prepared activated carbons. Kinetic and isotherm analysis declare that the sorption process is fitting well with pseudo second order kinetic model and Langmuir isotherm model. Thermodynamic analysis explores that U(VI) sorption using carbon A and B is exothermic, spontaneous, and feasible sorption process. The displayed results declare that the sorption capacity of Carbon A and B are 36.2 and 38.9 mg g^−1^ respectively, which indicates that the pyrolysis temperature improves the sorption characteristics of the yield sorbent materials.

## Electronic supplementary material

Below is the link to the electronic supplementary material.

Supplementary file1 (DOCX 159 KB)

## Data Availability

The datasets used and/or analyzed during the current study are available from the corresponding author on reasonable request.
