# Supplementary material for: Active carbon-based waste packaging materials for uranium sorption from aqueous solution
Source: Environ Sci Pollut Res Int. 2023 May 11;30(30):74726–41. doi: 10.1007/s11356-023-27269-7 (PMC10293414; doi:10.1007/s11356-023-27269-7)
Supplement: Supplementary file 1 — Supplementary file1 (DOCX 159 KB) [file 11356_2023_27269_MOESM1_ESM.docx]

**Supplementary Information**

**Figure S1:** *U(VI) speciation as a function of solution pH, calculated using Hydra-MEDUSA equilibrium calculation program* ***(Hussein and Taha 2013)****.*

**Reference:**

*Hussein AEM, Taha MH (2013) Uranium removal from nitric acid raffinate solution by solvent immobilized PVC cement. J Radioanal Nucl Chem 295:709–715. https://doi.org/10.1007/S10967-012-2158-3/TABLES/2*

*
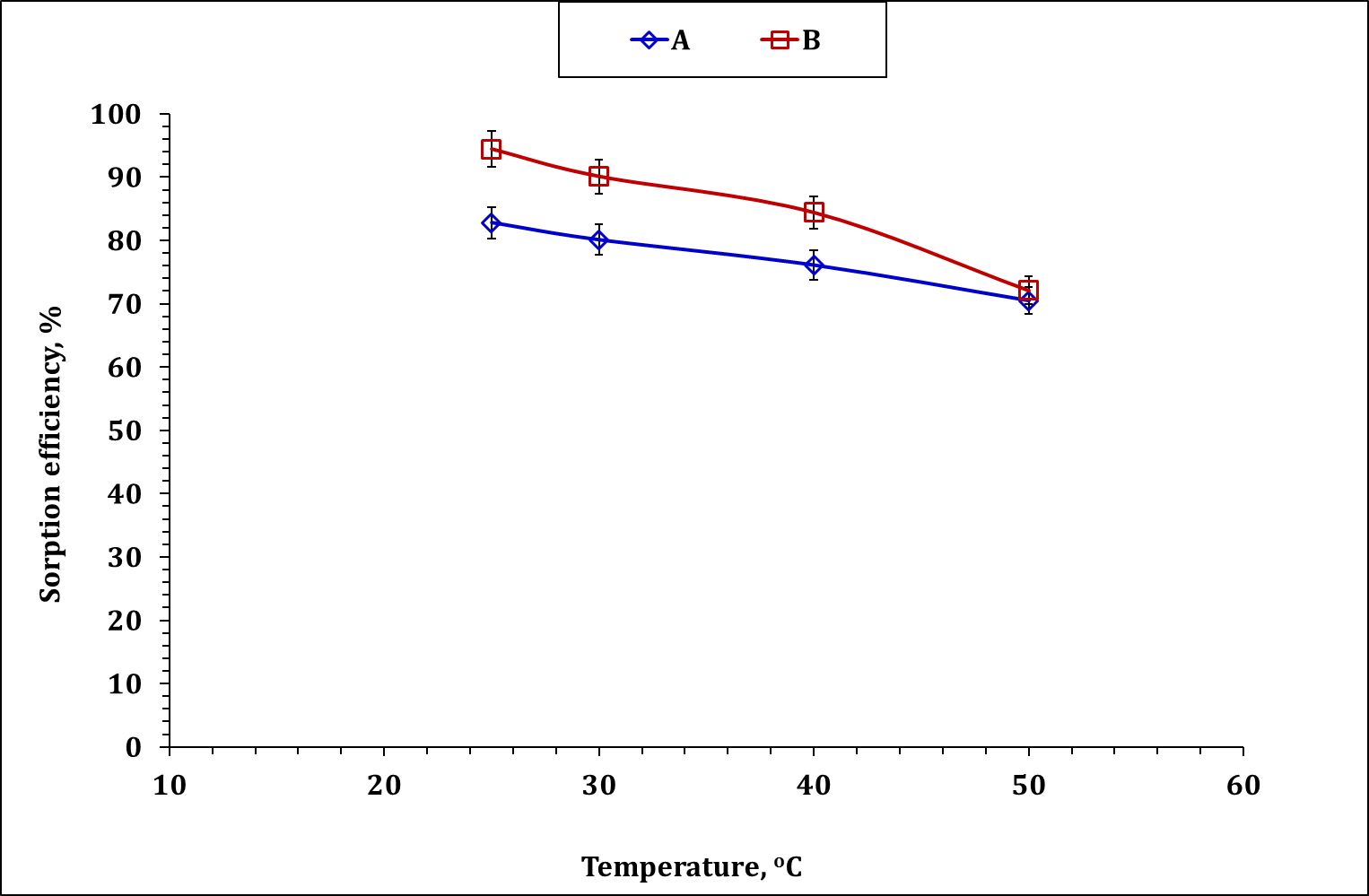
*

**Figure S2:** *The sorption efficiency of U(VI) as a function of temperature.*


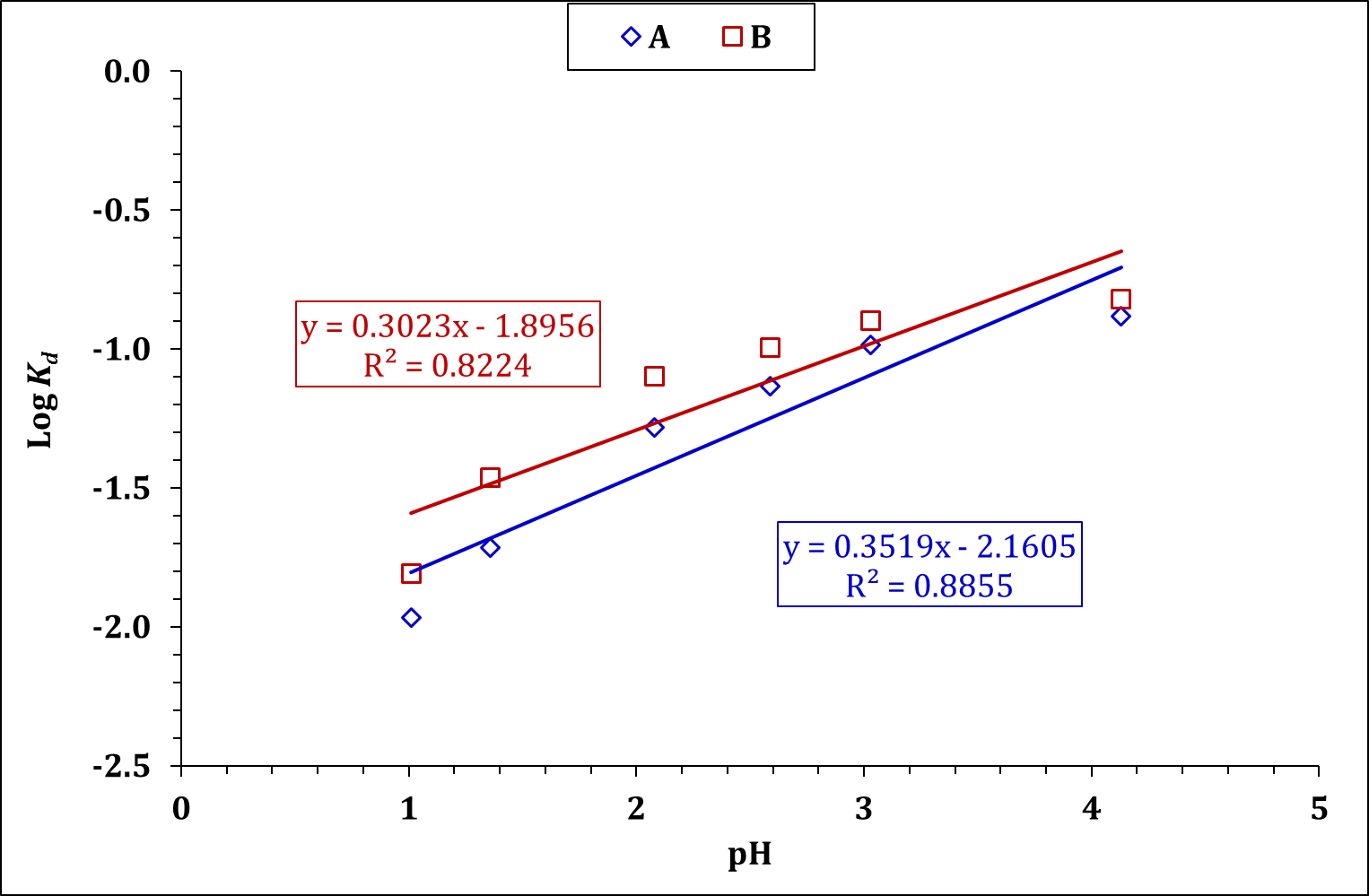


**Figure S3:** *Variation of log Kd as a function of solution pH.*

*.*


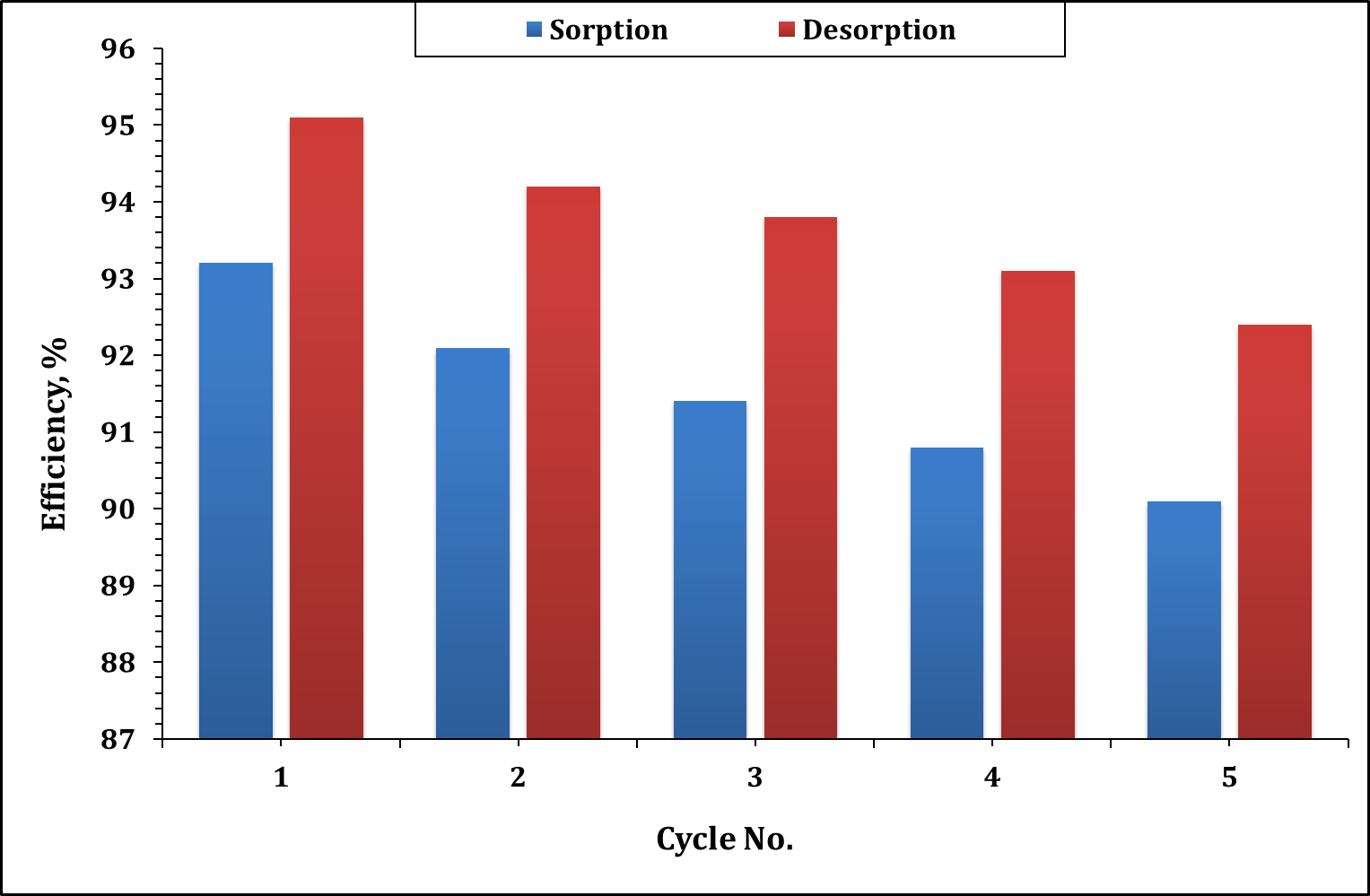


**Figure S4:** *Sorption and desorption cycles.*

**Table S1**: Kinetics and isotherm models for U(VI) sorption using carbon A and B **(Marques et al. 2019; Kang and Kim 2019)**.

| Kinetics | Equations |
| --- | --- |
| Pseudo-first-order | $q_{t}=q_{1}\left( 1-e^{-k1t} \right)$ |
| Pseudo-second-order | $q_{t}=\frac{1}{\left( 1 \vert k_{2}q_{2}^{2} \right)+\left( t \vert q_{2} \right)}$ |
| Intra-particle diffusion model (IPD) | $q_{t}= K_{id}t^{0.5} + C_{i}$ |
| Isotherms | **Equations** |
| Langmuir model | $q_{e}=\frac{q_{m}k_{L}C_{e}}{1+k_{L}C_{e}}$ |
| Freundlich model | $q_{e}=K_{F}C_{e}^{1/{n_{F}}}$ |
| Sips model | $q_{e}=\frac{q_{S}\left( k_{S}C_{e} \right)^{mS}}{1+\left( k_{S}C_{e} \right)^{mS}}$ |
| Fitting Equations | **Equations** |
| Coordination coefficient ($\boldsymbol{R}^{\boldsymbol{2}}$) | $R^{2}=1-\frac{\sum_{1}^{X} \left( {q_{exp}-q}_{pred} \right)^{2}}{\sum_{1}^{X} \left( q_{exp}-\bar{q_{exp}} \right)^{2}}$ |
| Chi-square coefficient ($\boldsymbol{x}^{\boldsymbol{2}}$) | $x^{2}=\sum\left[ \frac{\left( {q_{exp}-q}_{pred} \right)^{2}}{q_{pred}} \right]$ |

qe (mg g^-1^) is the equilibrium concentration of U(VI) ions, and qt (mg g^-1^) is the adsorbed amount of U(VI) ions after time t (min), Ce (mg L^-1^) is equilibrium concentration of U(VI) ions. k_1_ (min^-1^) and k_2_ (min^-1^) are the rate constants for the pseudo first and second order, respectively. K_id_ (mg/g. min^0.5^) is a rate constant, and C is the thickness of the boundary layer. q_m_ and q_S_ are the maximum sorption capacity (mg. g^-1^) of Langmuir and Sips models. k_L_ (L. mg^-1^), K_F_ (L/ mg), and K_S_ (L/ mg) are represent the constants of Langmuir, Freundlich, and Sips models, n refer to the sorption intensity, mS is Sips constant. $R^{2}$ and $x^{2}$ are the coordination and Chi-square coefﬁcients respectively, the experimental equilibrium capacity is $q_{exp}$ (mg g^-1^), while the predicted capacity is $q_{pred}$ (mg g^-1^), and X is the number of test points.

**Reference:**

*Kang HJ, Kim JH (2019) Adsorption Kinetics, Mechanism, Isotherm, and Thermodynamic Analysis of Paclitaxel from Extracts of Taxus chinensis Cell Cultures onto Sylopute. Biotechnol Bioprocess Eng 24:513–521.* [*https://doi.org/10.1007/s12257-019-0001-1*](https://doi.org/10.1007/s12257-019-0001-1)

*Marques BS, Frantz TS, Sant’Anna Cadaval Junior TR, et al (2019) Adsorption of a textile dye onto piaçava fibers: kinetic, equilibrium, thermodynamics, and application in simulated effluents. Environ Sci Pollut Res 26:28584–28592.* [*https://doi.org/10.1007/s11356-018-3587-5*](https://doi.org/10.1007/s11356-018-3587-5)

**Table S2:** *The evaluated parameters of* Morris-Weber model kinetic model

**Table S3:** U(VI) desorption percent *using different solutions.*

| Solution | Concentration, M | E, % |
| --- | --- | --- |
| HNO_3_ | 1.0 | 69.2 |
|  | 0.5 | 57.7 |
| H_2_SO_4_ | 1.0 | 95.2 |
|  | 0.5 | 86.5 |
| HCl | 1.0 | 63.5 |
|  | 0.5 | 51.9 |
